# Hybrid Polymer Composites Based on Polystyrene (PS) Used in the Melted and Extruded Manufacturing Technology

**DOI:** 10.3390/polym14225000

**Published:** 2022-11-18

**Authors:** Katarzyna Bulanda, Mariusz Oleksy, Rafał Oliwa

**Affiliations:** Department of Polymer Composites, Faculty of Chemistry, Rzeszow University of Technology, Al. Powstańców Warszawy 6, 35-959 Rzeszów, Poland

**Keywords:** polystyrene (PS), hybrid polymer composites, rapid prototyping (RP), modelling, characterization, optimal design

## Abstract

As part of the work, innovative hybrid polymer composites dedicated to rapid prototyping, especially for 3D printing with the melted and extruded manufacturing (MEM) technique, were developed. For this purpose, the influence of modified fillers, such as alumina-modified silica, bentonite modified with quaternary ammonium salt, and lignin/silicon dioxide hybrid filler, on the functional properties of polystyrene-based composites was investigated. The introduced additives were selected to improve the processing properties of polystyrene (PS), in particular its thermal stability, while maintaining good mechanical properties. In the first part of the work, using the proprietary technological line, filaments from unfilled PS and its composites were obtained, which contain modified fillers in the amount of 1.5% to 3.0% by weight. Samples for testing functional properties were obtained by 3D printing in MEM technology and injection technique. The rheological properties—mass melt flow rate (MFR), viscosity, and mechanical properties—are presented in the further part of the work. The size and the respective dispersion in the polystyrene polymer matrix of the fillers used were determined by scanning electron microscopy with energy dispersion spectroscopy (SEM/EDS). The correct dispersion of additives in PS was also confirmed by wide-angle X-ray analysis (WAXS). A significant improvement in the thermal stability of the obtained composites after the introduction of fillers into the polymer matrix was confirmed on the basis of thermogravimetric analysis (TGA). The remaining tests of physicochemical properties, differential scanning calorimetry (DSC), and infrared spectroscopy with Fourier transform (FT-IR) allowed us to state no significant changes in relation to polystyrene. The obtained test results allowed us to conclude that the amount and type of fillers used in the PS polymer matrix significantly affect the performance properties of the tested hybrid composites. The composites obtained as part of the work can be successfully used in rapid prototyping technologies, especially for the production of details originally designed from PS, which are required to have higher thermal stability than is guaranteed only by the polymer matrix.

## 1. Introduction

**Figure 1:**
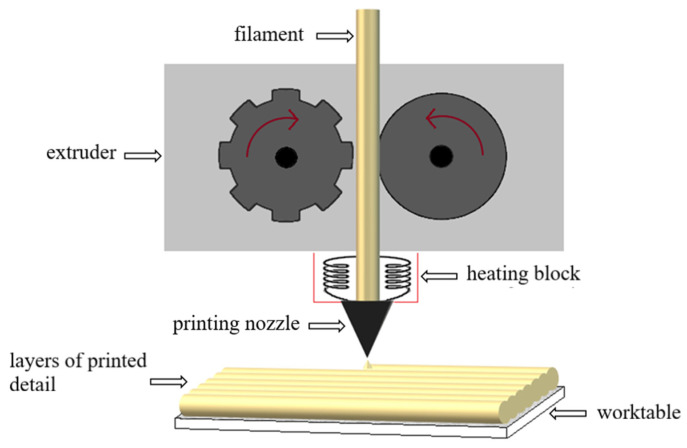
3D printing process in MEM technology.

Currently, additive manufacturing (AM) technologies, due to the possibility of producing difficult geometries combined with short lead times, have become very attractive for many industries [1,2,3,4]. The main advantages of this process are low costs of equipment and its operation and maintenance, low operating temperature, and a small amount of waste [5,6]. The techniques that combine the advantages of AM are primarily methods of extruding material (ME), including melted and extruded manufacturing (MEM). The ME technology, printing with molten filament, is one of the older 3D printing techniques [7,8,9,10,11,12]. It was originally developed for design applications and the creation of functional prototypes, but now it has gained considerable recognition in the industry due to its repeatability, efficiency, low cost, and the ability to produce geometrically complex shapes to produce parts from commonly used, popular thermoplastics [13,14,15,16,17]. MEM technology (Figure 1) enables the production of 3D structures by depositing materials in a layer-by-layer manner based on a series of thin cross-section images provided by computer-aided design (CAD) [4,13,14,15,16,17]. The material used is in the form of a filament, which allows for constant dosing of the material into the melting system chamber [17].

While obtaining a single layer, the head moves in the XY plane. The next layer is applied after the build plate has been lowered by a given layer thickness value. The printing process is controlled directly from the printing machine, and by computer through software specialized for a given 3D printer [15,16,17].

At the beginning, AM was focused only on polymers whose mechanical properties still did not match those made with standard methods, such as injection molded. For this reason, polymers have been replaced or supplemented by composites, ceramics, and metals. Polymer matrix composites are preferred in industry because they meet high requirements for mechanical and thermal performance with close dimensional tolerances, even over a long service life [1,7,8,9,10,11,12,13]. However, the library of polymeric materials available for AM is still limited [1].

Despite the advantages of AM technology, there are only a few studies in the open literature that investigate the functional properties of polystyrene-based (PS) composites dedicated to 3D printing [18]. Typically, high impact polystyrene (HIPS) is used in ME technologies. However, HIPS requires high printing temperatures, as the nozzle temperature should reach 230–240 °C and the heatbed should reach 90–100 °C. The material is usually used to support elements, and in order to dissolve them, a limonene solution is used [19,20]. However, PS may become a polymer with great potential for 3D printing, as it is characterized by high dimensional stability, low cost, and is easy to process and dye [21,22].

To sum up, there are only a few researchers focused on the modification of the functional properties of polystyrene intended for applications in the melted and extruded manufacturing technology, which is why the authors of this paper decided to fill this gap. Regarding PS, due to its numerous advantages, polystyrene is a colorless material (easy to dye in different colors) and hard, has a low softening point and low viscosity compared to standard thermoplastics such as polyethylene or polypropylene, is a very interesting material, and easily processes rapid prototyping methods. Therefore, as part of this work, hybrid polymer composites based on PS with the addition of selected modified nanofillers and fillers were developed and obtained. The introduced additives were selected so as to improve the processing properties of PS, in particular its thermal stability, while maintaining good mechanical properties. The polymer composites obtained in this way significantly increase the range of materials intended for 3D printing, and the tests and analysis of the results allow us to present the full characteristics of the composites produced. The research presented in the article is a continuation of other works [23,24]. In the future, the composites obtained in this way will be used for the production of selected machine elements in the technology of rapid prototyping and injection molding.

## 2. Materials and Methods

### 2.1. Materials

Commercial granules (Total 1160, Total-Chem Sp.z o.o., Kraków, Poland) marked as PS were used as the polymer matrix. PS was filled with silica (S) containing alumina (Aerosil MOX 170, Evonic Industries, Hanau, Germany), bentonite (B) (technical product “Specjal” Zębiec SA Zakłady Górniczo-Metalowe, Zębiec, Poland) modified with quaternary ammonium salt (BARQUAT^®^ DM80, Lonza, Switzerland), and lignin (L) (Sigma-Aldrich, St. Louis, MO, USA)/silicon dioxide (Syloid 244, WR Grace & Co., Columbia, MD, USA) hybrid filler. Detailed information on the procedure of obtaining bentonite modified with quaternary ammonium salt and lignin/silicon dioxide filler has already been patented and described earlier [25,26]. Modified fillers were introduced into PS to improve the physicochemical properties, especially thermal stability, while maintaining or improving the mechanical properties. Hybrid filler systems were introduced into the polymer in order to investigate the synergy effect of their action and the impact on the functional properties of the obtained composites. Chemically modified polyethylene grafted with maleic anhydride (Fusabond E926, DuPont, Wilmington, DE, USA) was used as a compatibilizer.

The compositions of the individual compositions are summarized in Table 1.

### 2.2. Preparation of the Composite and Sample

The individual materials were dried in a vacuum oven (Colect, Krakow, Poland) before starting the process (PS: 80 °C, 3 h; fillers S, B, L: 100 °C, 24 h). Then, the components of the composition in appropriate amounts were homogenized on a Coperion twin-screw extruder, which is equipped with a granulation line, process parameters: screw speed 400 rpm, extrusion capacity 4 kg/h, temperature range 220–250 °C. The obtained granules were dried in a vacuum oven at 80 °C for 3 h. The obtained polymer composites were used to obtain fibers with a diameter of approximately 1.75 ± 0.05 mm on the designed filament production line (Gamart SA, Jasło, Poland) in the temperature range 210–235 °C.

The designed proprietary technological line is presented elsewhere [23,24].

**Figure 2:**
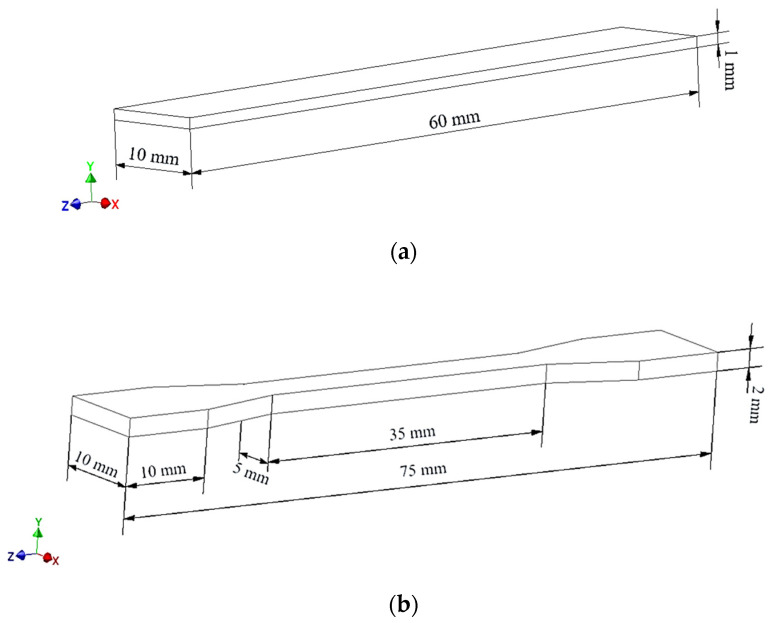
The dimensions of the samples, respectively: (**a**) a bar and (**b**) a paddle.

The composites were used to obtain samples (Figure 2) needed for further tests on the 3D printer (UP BOX, TierTime, Beijing, China) in the MEM technology and by the injection method on the Haake MiniJet II mini-injection molding machine (Waltham, MA USA).

The process parameters are summarized in Table 2.

### 2.3. Methods Characterization

Mass flow rate tests were performed using a plastometer (DYNISCO 4781, Kayeness INC., Honey Brook, PA, USA). The determination consisted of introducing a sample weighing 4 g into the apparatus heated to the processing temperature (250 °C) and plasticizing it within 240 s using a preload equal to 1.1 kg. The actual measurement was carried out with a load of 2.16 kg. After the preset time (10 s), the extruded sample was cut off and weighed after it had cooled down. For each composite, three measurements were made in accordance with the guidelines of ISO 1133 [27].

Viscosity was determined using a capillary rheometer (Smart RHEO, Instron Ceast, Norwood, MA, USA). The determination consisted in introducing the sample (10 g) into an apparatus heated to the processing temperature PS (250 °C) equipped with a capillary (length 40 mm, width 1.16 mm), where the sample was thermostated during 300 s using a preload. The shear rate was set in the range of 250–2000 1/s. The determination was made in accordance with the recommendations of the standard ISO 11443: 2005 [28].

Rockwell hardness was determined using a durometer (Zwick/Roell, Zwick GmbH & Co., Ulm, Germany). The test sample was placed in a durometer, then a load was applied, controlling the height of the indentation (0.15–0.35 mm), and the measurement was started. Ten determinations were carried out for each material according to the recommendations of ISO 6508 [29].

Charpy impact strength was determined with a hammer with a force of 1 J (PSW GEHARD ZORN, Stendal, Germany). Before the measurement, a notch (CEAST-Instron, Pianezza TO, Italy) was cut to a width of 2 mm on each sample. The samples were placed in the apparatus in a horizontal edge position in such a way that the hammer hit the very center of the bar edge. For each material, five measurements were made according to the recommendations of the ISO/179/1Ea standard [30].

The mechanical properties were determined in a static tensile test, which was carried out with a testing machine (INSTRON 5967, Grove City, PA, USA). For this purpose, the test specimens in the shape of blades were placed in the machine holders and the measurement was started. At a tensile speed of 5 mm/min, the Young’s modulus was determined, and after obtaining a 1% tensile strain, the speed was increased to 50 mm/min. Five measurements were made for each material according to the recommendations of ISO 527 [31].

To observe the microstructure, brittle fractures obtained by impact fracture of the sample after cooling it in liquid nitrogen were used. The observations were made using the scanning electron microscope (Hitachi TM3000, Red Star Vietnam Co., Ltd., Hanoi, Vietnam) with an energy dispersive spectroscopy, EDS microanalysis device. Before the observations, samples of brittle fractures of polymers and composites were sputtered with a layer of gold with palladium. The observations were made using a voltage of 5 keV.

Observations of the microstructure were performed using a scanning electron microscope, SEM (Hitachi TM3000, Red Star Vietnam Co., Ltd., Hanoi, Vietnam) with energy dispersion spectroscopy, EDS. Brittle fractures of the tested samples were observed, which were obtained after placing the sample in liquid nitrogen and then impact fracture. The samples were sprayed with a layer of gold and palladium. The observations were made using a voltage of 5 keV.

Thermogravimetric analysis (TGA) was performed under a nitrogen atmosphere using a TGA/DSC1 apparatus (Mettler Toledo DSC1 STARᵉ System, METTLER Toledo, Schwerzenbach, Switzerland). The samples weighing approximately 5 mg were tested on 25–600 °C platinum plates at the rate of 10 °C/min. The obtained results were analyzed using the STAR^e^ software.

Differential scanning calorimetry (DSC) studies were also carried out in a helium atmosphere with an apparatus (Mettler Toledo DSC 1 Starᵉ System, METTLER Toledo, Schwerzenbach, Switzerland). Measurements were made in a helium atmosphere in airtight aluminum crucibles. Samples weighing approximately 6 mg of samples were placed in aluminum crucibles and tested in the range of 0–300 °C at a speed of 10 °C/min, cooled to 0 °C at a speed of 10 °C/min and heated again to a temperature of 300 °C with speed 10 °C/min.

Wide-angle X-ray diffraction (WAXS) testing was performed with a diffractometer (NanoStar-U, Bruker Inc., Billerica, MA, USA), which is equipped with a two-dimensional detector in transmission geometry. X-rays (1.54 Å) were produced by irradiating a copper lamp, which was supplied with a voltage of 600 µA at 50 kV. The scattering angle range used was in the range of 0°–28°.

Fourier transform infrared spectroscopy (FTIR) was also performed using a Nicolet 8700 spectrophotometer, which was equipped with a diamond ATR attachment. The wavelength was 4000–650 cm^−1^, and each material was scanned 128 times. The obtained results were analyzed with the use of specialized OMNIC Spectra software (Thermo Scientific™, Waltham, MA, USA, Stany Zjednoczone).

## 3. Results and Discussion

The knowledge of the rheological data of the tested materials is important in terms of proper design and proper processing. Table 3 summarizes the results of the determined mass flow rate (MFR) of the obtained polystyrene-based composites. Only in the case of the PS/3%S composite was the material flowability higher (8.37 g/10 min) compared to the PS (7.87 g/10 min). In other cases, the results were slightly lower by about 5.84–3.81%.

The observed decrease in the MFR value after the introduction of the modified fillers into the PS matrix is so small that it did not adversely affect the properties of the molding process, both by injection and 3D printing.

**Figure 3:**
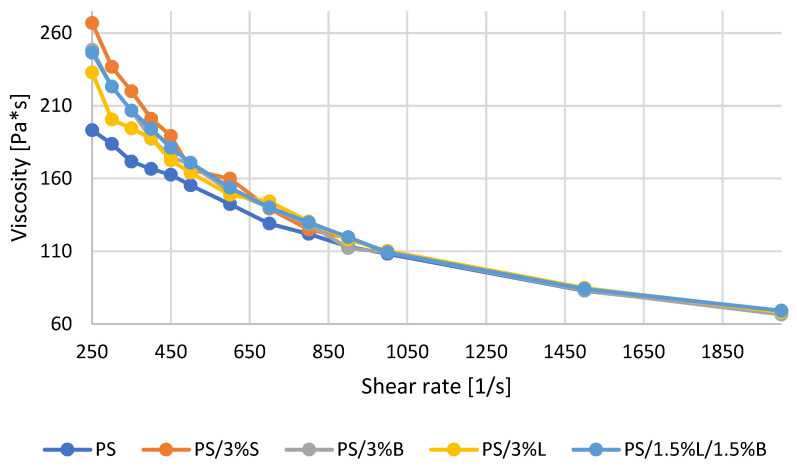
Viscosity curves of PS polymer and composites based on PS.

The results of determination of the flow curves for polystyrene and composites on the PS matrix are summarized in Figure 3 in the form of a graph. It can be seen that the viscosity of the materials decreases with increasing shear rate, which corresponds to the behavior of the non-Newtonian fluid during the extrusion process. The reason is that the increase in the shear rate is helpful in unraveling and aligning the polymer chains in the flow field, which additionally results in less flow resistance and a decrease in viscosity [32,33]. The addition of modified fillers increased the viscosity results of the obtained composites only within a certain range. Only at low values of 250–500 s^−1^ was the difference in viscosity of individual composites noticeable, and the lowest result at 250 s^−1^ was obtained for PS 195.52 Pa*s, then PS/3%L, PS/1.5%L/1.5%, PS/3%B, while the highest result was obtained for PS/3%S 266.8 Pa*s. At a shear rate of 1000 s^−1^ and higher, all tested materials had almost the same viscosity. This is a general phenomenon that occurs when chains are disentangled at high shear rates [33].

Samples obtained by 3D printing methods generally have worse mechanical properties than samples obtained by additive production. This observation is widely described in the literature [34,35,36] and results from a greater homogeneity of samples obtained by injection methods. For this reason, it was decided to present the obtained research results separately using both techniques.

**Figure 4:**
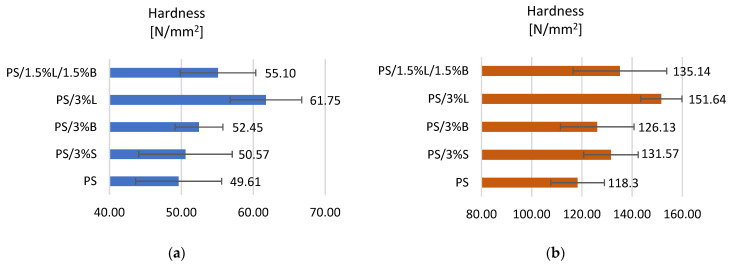
Hardness test results of (**a**) samples obtained by 3D printing, (**b**) samples obtained by injection molding.

Figure 4a summarizes the results obtained for polystyrene and the composites obtained on its matrix. The introduction of modified fillers to the polymer resulted in an increase in the hardness of the material. When analyzing the results obtained for the MEM technology fittings, it was observed that PS/3%L is characterized by the highest hardness, 24.47% higher compared to the unmodified polymer (49.61 N/mm^2^). Composite shapes obtained by injection molding are characterized by better hardness (Figure 4b) compared to composite shapes obtained by 3D printing (Figure 4a). The introduced fillers S, B, L, and L/B allowed us to obtain higher hardness values in the range from 126.18–151.64 N/mm^2^. The composite with the addition of 3%L has the best hardness, and its hardness increased by 28.18% compared to the unfilled polymer. Composites containing modified lignin in the composition are characterized by an increase in hardness, because the viscosity of the material increased after the filler was introduced into the polymer matrix, which allowed us to obtain more homogeneous shapes (precise printing and shape filling). Similar results were obtained by other scientists [37,38,39] by introducing up to 5% by weight of the filler, and higher contents may cause the formation of agglomerates and/or aggregates of the additive, which significantly reduces the tested parameter.

**Figure 5:**
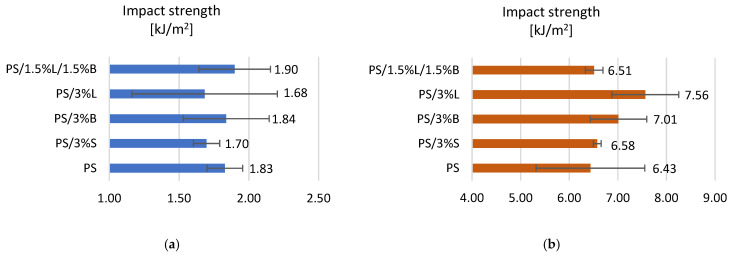
Impact strength test results of (**a**) samples obtained by 3D printing, (**b**) samples obtained by injection molding.

The results of the impact strength measured according to Charpy for polystyrene and composites on its matrix are summarized in Figure 5. For composite shapes made with a 3D printer, the addition of modified bentonite and lignin (PS/3%B and PS/1.5%L/1.5%B) improved the impact toughness because the results for composites were 1.84 kJ/m^2^ and 1.90 kJ/m^2^, respectively. For the remaining composites, a slight deterioration of the impact toughness by about 7% was observed. On the other hand, for composite moldings obtained in the technology of injection into the mold (Figure 5b), in each case the impact strength was higher compared to PS (6.43 kJ/m^2^), even up to 17.57%.

The results of testing the strength properties for polystyrene and composites on the PS matrix were collected and presented in Table 4. Taking into account the results obtained for fittings made with additive manufacturing for composites on the PS matrix, it was noticed that after adding fillers, Young’s modulus was slightly decreased by a maximum of 9.16% compared to the unmodified polymer matrix. Only for PS/3%L was the obtained result higher than PS, equal to 2063.41 MPa, and it should be mentioned here that this effect is related to the increase of composite hardness (61.75 N/mm^2^), which was discussed in the previous section. Moreover, in the case of testing the moldings obtained by the injection molding method, a similar tendency was observed, because for composites with the addition of modified fillers, lower values of Young’s modulus were obtained compared to PS.

The results of tensile stress and strain at breaks obtained for fittings made with 3D printing, summarized in Table 4, show that the introduced fillers had a negative impact on the values of the parameters tested. The obtained composites are characterized by slightly lower breaking stress and strain at break compared to the polymer matrix. The worst results were obtained for PS/3%L. This effect is probably related to the increase in stiffness and brittleness of the obtained composite. Analyzing the subsequent data in Table 4 for the moldings obtained by the injection method, an analogous relationship was observed in the obtained test results as in the case of composites obtained by the 3D printing method.

**Figure 6:**
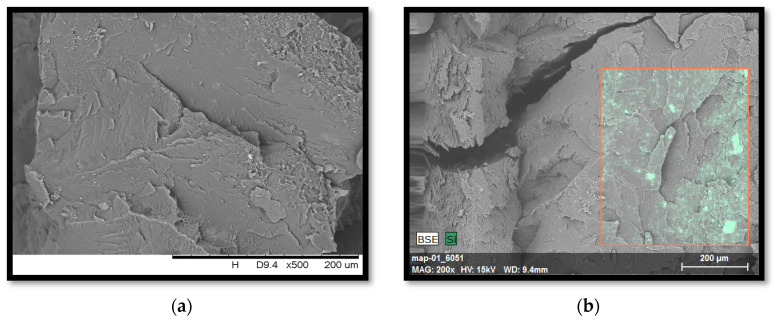

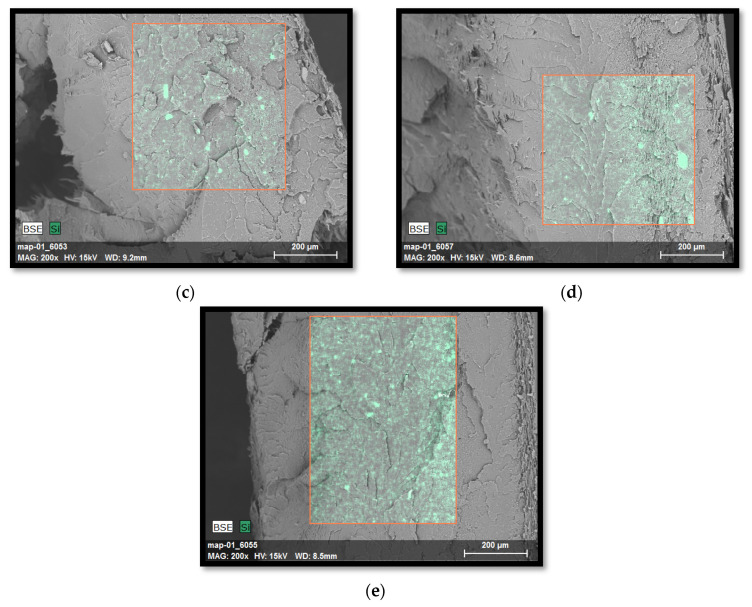
SEM micrographs with an EDS attachment of PS polymer and composites based on PS: (**a**) PS, (**b**) PS/3%S, (**c**) PS/3%B, (**d**) PS/3%L, (**e**) PS/1.5%L/1.5%B.

Brittle fractures of polystyrene and composites on its matrix were subjected to SEM imaging with an EDS analyzer (Figure 6). The unfilled polymer matrix is characterized by the presence of a few grooves on the surface. The introduced modified fillers resulted in the formation of a greater number of surface-uneven protrusions. It is not possible to distinguish the phases, therefore the area subjected to EDS analysis was marked in red, and the selected element was silicon, which was present in each type of filler used (Figure 6b–e). Analyzing the obtained micrographs, a good distribution of additives in the polymer matrix of polystyrene was found. In the presence of modified silica, bentonite, and lignin, few filler clusters were visible. The best dispersion was obtained for the PS/1.5%L/1.5%B composite containing a hybrid system of lignin and bentonite, which directly translated into the previously presented good mechanical properties of the material.

**Figure 7:**
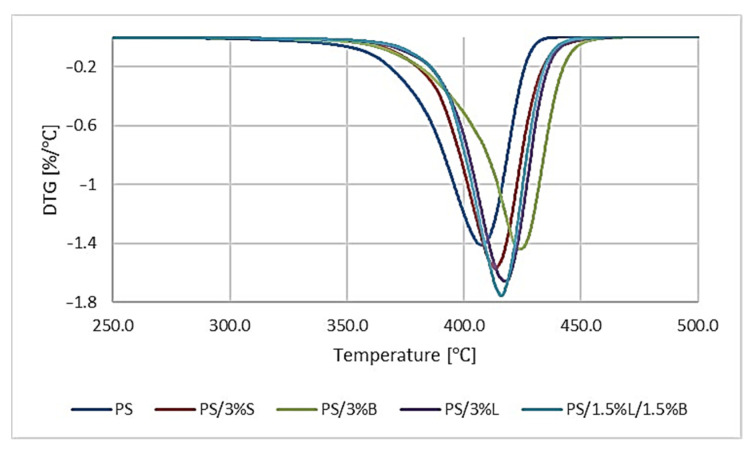
Results of mass change derivative curve analysis of PS polymer and composites based on PS.

The results of testing the thermal stability properties of the composites are summarized in Figure 7 and Table 5. The temperature of 5% weight loss (T_5%_) was determined from the TGA curve, which can be taken as the beginning of the degradation process. The maximum temperature of the degradation steps (T_1_) was determined from the mass change derivative curve.

Thermogravimetric analysis also included PS and composites on its matrix. Observing the obtained research results (Figure 7), we found that the materials were characterized by a single-stage intense peak in the temperature range of 325–465 °C. For unmodified PS, the lowest temperatures of volatile substances loss (T_2%_), the beginning of the degradation process (T_5%_), and the maximum temperature of the degradation stage (T_1_) were obtained. The dominant mechanisms of thermal degradation of polystyrene are chain breakage and diffusion of free radicals. Therefore, the introduced additive particles can suppress the mobility of the PS chain [40]. The introduced modified fillers increased the thermal stability of the polymer (Table 5), and the best thermal stability results were obtained for composites containing modified lignin in the composition. For PS/3%L and PS/1.5%L/1.5%B, the beginning of the degradation process was read at 393.62 °C and 393.41 °C, which is an increase of the tested parameter by 18.75 °C and 18.54 °C compared to unfilled polymer matrix. Composites degrade at higher temperatures because functional cleavage in the lignin chains occurs at higher temperatures, resulting in residual aromatic moieties forming efficient carbon. The formed layer creates a protective layer as it reduces the effective heat transfer between the gas phase and the condensed phase [31]. A much better thermal stability of PS was also observed after the introduction of modified silica or modified bentonite into the matrix. In the case of PS/3%S, this may be related to the relatively large specific surface area of the silica filler. The presence of 3%S in the polymer matrix leads to the formation of an interlayer on the filler surface, and thus to the immobilization of the polymer chains [41]. On the other hand, in the case of PS/3%B, the effect can be explained by the formation of a layered structure by modified bentonite plates obtained thanks to appropriate homogenization, which inhibits heat transfer in the polymer matrix [42]. For PS/3%S and PS/3%B the T_2%_ was higher by 15.33 °C and 14.62 °C compared to the unfilled polymer, while T_5%_ was higher by 12.78 °C and 13.23 °C.

**Figure 8:**
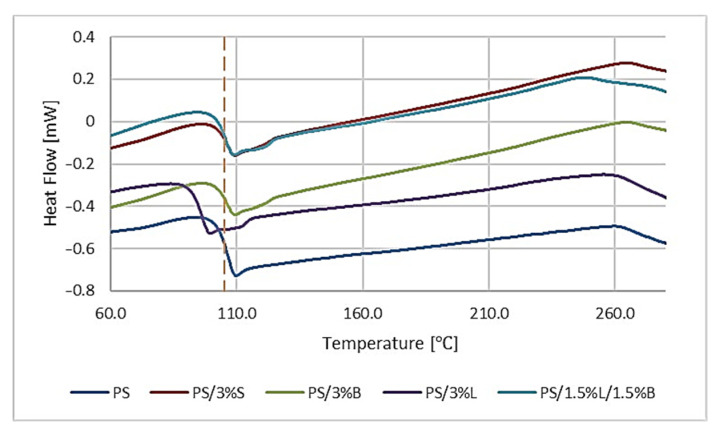
Results of the differential scanning calorimetry (DSC) analysis of PS and PS composites.

The deflection takes place at a temperature of about 100 °C, which corresponds to the glass transition temperature (Tg) PS [43,44]. The introduction of modified S, B, and L fillers and the hybrid L/B system did not cause significant changes in the thermal history of PS, because the Tg was obtained for composites in the range of 104.77 °C for PS/3%B–105.94 °C for PS/3%S, while for the unfilled polymer matrix it was exactly 105.05 °C (Figure 8).

**Figure 9:**
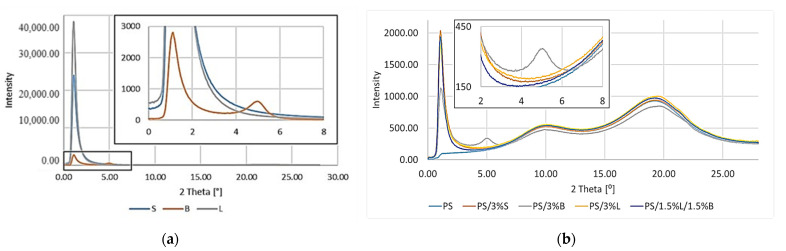
WAXS patterns: (**a**) fillers; (**b**) PS and composites with the addition of modified S, B, and L fillers.

The morphology and molecular orientation of the composites and fillers were characterized by WAXS analysis, and the plots of radiation intensity are a function of scattering angle on Figure 9a,b.

The scattering angle for the tested materials was in the range from 0° to 28°. Due to the capabilities of the apparatus for fillers in this range, it was only possible to observe the peak for the modified bentonite B (Figure 9a).

The distance between successive planes of the filler (d_hkl_) was calculated from the Bragg formula (Equation (1)):

(1) $$d_{\mathrm{hkl}}=\frac{n\lambda }{2sin\theta },$$

where: *n* is the degree of diffraction (*n* = 1, 2…), *λ* is the wavelength of radiation used, and 2*θ* is the angle at which the diffractive peak occurs, as read from the WAXS graph.

The particle size in the Scherrer formula (Equation (2)) was also determined:

(2) $$D_{\mathrm{hkl}}=\frac{K\lambda }{bcos\theta },$$

where: D_hkl_ is the reflex width dependent on the size of crystallites, *K* is Scherrer’s permanent, *K* = 1, λ is the wavelength of radiation used, and b is the half-width of the diffraction peak for the plane (_hkl_).

Analyzing the results obtained for the fillers (Figure 9a), a peak for B at 4.98° can be seen, which can be attributed to the diffraction reflection from the bentonite (001) sheets [45]. Unfortunately, it was not possible to determine the characteristic peaks for the remaining modified S and L fillers due to the applied assay conditions and technical capabilities of the apparatus. The distances between successive packets of filler plates and the size of their particles were calculated. For B d_khl_ it was 18.20 Å, while for D_khl_ it was 110.8 Å.

Tests were carried out using wide-angle X-ray diffraction of polystyrene and composites on its matrix (Figure 9b). By analyzing the obtained test results, it was found that all the obtained polymer materials are characterized by the presence of two wide, low-intensity peaks at 2θ of approximately 10° and 19°. The bands obtained were assigned to the PS polymer, which was also confirmed by the literature data [46]. The introduced modified fillers were well-dispersed in the polymer matrix, because an almost identical course of the curves was obtained for the composites and the polymer matrix. In the case of PS/3%B there was an additional peak at 2θ of 5°, which was attributed to the modified bentonite. The results of the WAXS test for the fillers also revealed the presence of the B additive in this range. It can be concluded that the filler particles were not properly spaced apart, and thus the PS/3%B is not characterized by an adequate filler dispersion in the PS matrix.

**Figure 10:**
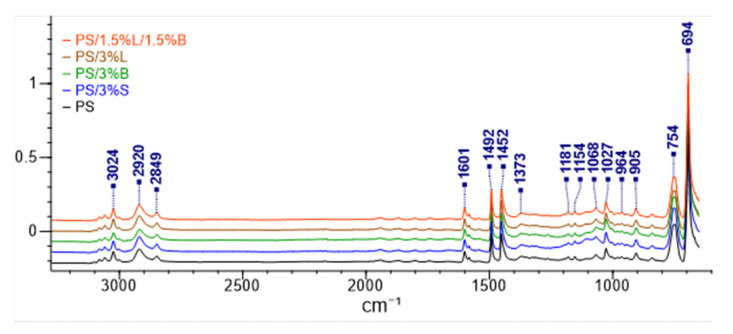
FT-IR spectra recorded for the composition.

In the next stage of the work, the chemical bonds of polystyrene and composites on its matrix were characterized (Figure 10). All spectra are characterized by bands characteristic for the polymer. No other additional peaks, which could be derived from chemical bonds of the introduced fillers, were determined. Peaks at 3024 cm^−1^, 2920 cm^−1^, and 754 cm^−1^ were observed, which can be attributed to the C-H stretching vibration of the aromatic ring. On the other hand, the peak at 2849 cm^−1^ corresponds to the aliphatic C-H bond, and for the wavelengths 1601 cm^−1^, 1492 cm^−1^, and 1452 cm^−1^, and C-C stretching bonds of the aromatic ring were assigned. In contrast, the peak at 694 cm^−1^ is derived from benzoic ring bonds [47,48].

## 4. Conclusions

- Research was conducted on the development of hybrid polymer composites with a PS matrix with the addition of modified fillers dedicated to 3D printing in the melted and extruded manufacturing (MEM) technology. For this purpose, several fillers known and described in the literature were selected, dispersed in the polymer matrix, and then fibers were obtained on a specially designed and developed technological line. The influence of fillers on the properties of the obtained composites, including alumina modified silica, bentonite modified with quaternary ammonium salt, and the lignin/silicon dioxide hybrid filler system, was investigated.
- It was found that the addition of modified fillers to the PS matrix did not change the polymer flowability (MFR). The addition of modified fillers increased the viscosity results of the obtained composites only to a certain extent. At low values of 250–500 s^−1^ the difference in viscosity of individual composites is noticeable, and the lowest result at 250 s^−1^ was obtained for PS 195.52 Pa*s, while the highest result was obtained for PS/3%S 266.8 Pa*s.
- An increase in Rockwell hardness and Charpy impact toughness of the obtained composites was observed, both for injection molded and 3D printed samples. A decrease in material stiffness was also observed with the addition of modified fillers, as evidenced by the decrease in Young’s modulus for the samples, regardless of the production technique, with the exception of the PS/3%L (2063.41 MPa) sample obtained by 3D printing.
- The observations of the microstructure of composites using the SEM/EDS method confirmed the appropriate dispersion of additives in the PS polymer matrix and the nanometric size of the fillers, which was also observed on the basis of the WAXS analysis results.
- The TGA results show that the addition of fillers substantially increased the thermal stability of the composites. For unmodified PS, the lowest temperatures of volatile substances depletion (T_2%_ 357.6 °C), the beginning of the degradation process (T_5%_ 374.9 °C), and the maximum temperature of the degradation stage (T_1_ 415.3 °C) were obtained. The best thermal stability results were obtained for composites containing modified lignin. For PS/3%L and PS/1.5%L/1.5%B, the beginning of the degradation process was read at 393.62 °C and 393.41 °C, which is an increase of the tested parameter by 18.75 °C and 18.54 °C compared to unfilled polymer matrix. The DSC study showed that PS is characterized by phase transitions typical of the material, and the added additives did not change the thermal history of the composites. The obtained spectrum of the polymer (FT-IR) contains all the characteristic functional groups of polystyrene, and the introduced fillers did not affect the distribution of the bands obtained.
- The introduction of fillers had a positive effect on the processing properties of PS, in particular its thermal stability, while still maintaining good mechanical properties. The polymer composites obtained in this way significantly expand the range of materials intended for 3D printing, and the research and analysis of the results allow for the presentation of the full characteristics of the composites produced. In the future, polymeric materials obtained in this way will be used to produce selected machine elements in the technology of rapid prototyping and injection molding.

## Figures and Tables

**Table 1:** Compositional data of the composites.

Composition	PS Content (wt.%)	S Content (wt.%)	L Content (wt.%)	B Content (wt.%)	E926 Content (wt.%)
PS	100	-	-	-	-
PS/3%S	96	3	-	-	1
PS/3%B	96	-	-	3	1
PS/3%L	96	-	3	-	1
PS/1.5%L/1.5%B	96	-	1.5	1.5	1

**Table 2:** Selected printing and injection parameters.

Printing Parameters		Injection Parameters	Paddles	Bars
Nozzle diameter, mm	0.4	Mold temperature, °C	70	70
Layer height, mm	0.2	Injection temperature, °C	250	250
Infill percentage, %	100	Injection pressure, bar	600	650
Infill pattern, °	±45	Post pressure, bar	750	800
Extrusion temperature, °C	250	Plasticizing time, s	120	120
Bed temperature, °C	80	Injection time, s	5	5
Printing speeds, mm/s	70	Post time, s	3	3

**Table 3:** Summary of the obtained MFR results.

Composition	PS	PS/3%S	PS/3%B	PS/3%L	PS /1.5%L/1.5%B
MFR [g/10 min]	7.87 ± 0.01	8.37 ± 0.04	7.47 ± 0.02	7.57 ± 0.01	7.41 ± 0.08

± standard deviation.

**Table 4:** Results of mechanical tests of PS and composites based on PS.

Composition	Young’s Modulus [MPa]	Stress at Break [MPa]	Strain at Break [%]	Young’s Modulus [MPa]	Stress at Break [MPa]	Strain at Break [%]
	3D Printing			Injection		
PS	2046.62 ±70.07	39.53 ±3.17	2.63 ±0.03	2332.86 ±43.7	54.03 ±5.4	4.83 ±0.44
PS/3%S	1859.20 ±57.94	35.94 ±2.54	2.24 ±0.13	2323.19 ±37.2	53.99 ±2.74	3.23 ±0.09
PS/3%B	2026.80 ±53.34	39.51 ±0.74	2.34 ±0.15	2326.20 ±47.79	55.11 ±0.19	3.99 ±0.30
PS/3%L	2063.41 ±24.06	35.77 ±5.97	2.21 ±0.19	2360.72 ±39.38	54.10 ±4.42	2.87 ±0.56
PS/1.5%L/1.5%B	1973.54 ±60.42	37.55 ±0.39	2.26 ±0.06	2301.75 ±15.15	55.95 ±0.37	3.56 ±0.17

± standard deviation.

**Table 5:** The results of research on the properties of thermostability of composites.

Composites	T_2%_ (°C)	T_5%_ (°C)	T_1_ (°C)	ΔV_1_ (%/°C)	R_600_ (%)
PS	357.6	374.9	415.3	1.4	0.7
PS/3%S	372.9	387.7	419.5	1.6	4.0
PS/3%B	372.2	388.1	430.3	1.4	3.7
PS/3%L	379.6	393.6	424.2	1.7	2.8
PS/1.5%L/1.5%B	379.2	393.4	421.5	1.8	2.0

## Data Availability

The data presented in this study are available on request from the corresponding author.
